# Omission of Sentinel Lymph Node Biopsy in Early-Stage HER2+ and Triple-Negative Breast Cancer: A Retrospective Analysis

**DOI:** 10.3390/jpm15100501

**Published:** 2025-10-17

**Authors:** Amandine Causse d’Agraives, Rebecca Allievi, Raquel Diaz, Piero Fregatti

**Affiliations:** 1 Department of Surgical Sciences and Integrated Diagnostic (DISC), University of Genoa, 16132 Genoa, Italy; 2 Breast Surgery Unit, IRCCS Ospedale Policlinico San Martino, 16132 Genoa, Italy

**Keywords:** early-stage breast cancer, SLNB, elderly

## Abstract

**Background:** Sentinel lymph node biopsy (SLNB) is the standard procedure for axillary staging in early-stage breast cancer. However, its necessity for some patient groups is being reevaluated. This change mainly arises from the procedure’s impact on quality of life and new evidence suggesting that some patients can forgo it without affecting their overall survival. **Objective:** This study focuses on the omission of SLNB in elderly patients aged 80 and older with HER2-positive (HER2+) or triple-negative breast cancer (TNBC) who are clinically node-negative (cN0), comparing outcomes to other relevant studies. **Methods:** In this retrospective study, we analyzed 39 cN0 women aged 80 and older (mean age at surgery 85.8) with HER2+ or TNBC treated between 2016 and 2024. We assessed overall survival (OS), disease-free survival (DFS), and locoregional recurrence without performing SLNB. We used Kaplan–Meier estimates and Cox proportional hazards models to evaluate survival outcomes by subtype, tumor size, and Ki-67 index. **Results:** The median OS was 3.9 years (95% confidence interval [CI]: 3.1 years, not estimable [NE]); the 5-year OS was 43.4% (95% CI: 25.3–74.6). The 5-year DFS was 37.7% (95% CI: 21.5–66.2). The median follow-up was 36.5 months (approximately 3.0 years). Five recurrences (12.8%) and two complications (5.1%) occurred. Patients with TNBC had a 5-year OS of 58.2% compared with 35.9% in those with HER2+ disease (*p* = 0.414). Patients with a low Ki-67 index (≤25%) had a 5-year OS of 78.6% compared with 25.9% in those with higher Ki-67 (*p* = 0.080). Tumor size ≥pT2 was associated with a worse prognosis. **Conclusions:** In carefully selected elderly patients with HER2+ or TNBC and no clinical nodal involvement, omitting SLNB was not linked to significantly lower survival rates. The observed numerical differences according to Ki-67 and tumor size suggest that surgical de-escalation may be feasible in selected elderly patients to limit complications without compromising oncological safety.

## 1. Introduction

Sentinel lymph node biopsy (SLNB) is considered the gold standard for axillary staging in early-stage breast cancer as it indicates the need for axillary lymph node dissection (ALND) and adjuvant therapy [1]. While this method provides useful prognostic information, its benefits in early-stage breast cancer, especially for elderly patients, are limited, particularly for those at low risk of axillary lymph node metastasis [2].

Trials such as SOUND and CALGB 9343 have shown that, in carefully selected older women with hormone receptor–positive tumors, omission of SLNB does not compromise oncologic outcomes while reducing surgical burden [3,4]. These data have reinforced a broader tendency toward de-escalating axillary surgery in the elderly. At the same time, several studies suggest that tumor biology and comorbidities may be more relevant than nodal status in determining survival in this population [5].

This perspective is especially relevant given the potential risks associated with the procedure. Patients undergoing SLNB are exposed to complications such as lymphedema, seroma formation, wound infections, reduced arm mobility, and sensory disturbances. These complications, while often considered manageable, are not negligible, even among older cohorts, and can substantially impair the quality of life of potentially already frail patients [6,7].

Considering this evidence, it appears safe to say that there is a need for personalized, tailored surgical approaches and more generally toward individualized therapy in elderly breast cancer patients. The primary goal is to limit unnecessary procedures, reduce hospitalization time, and minimize treatment-related complications without compromising oncological results. However, certain high-risk subtypes, such as HER2+ and TNBC, are associated with a higher risk of recurrence [8,9,10,11], and patients with these diagnoses often continue to undergo axillary staging.

In an effort to support a more balanced, patient-centered strategy, we retrospectively analysed outcomes in a cohort of very elderly women (≥80 years) with HER2+ or TNBC breast cancer treated surgically without SLNB or ALND. The aim of this exploratory study is not to provide definitive evidence but rather to generate preliminary data and contextualize results within the existing literature. In doing so, we seek to contribute to the ongoing discussion on tailored axillary management strategies for elderly patients with higher-risk subtypes.

## 2. Materials and Methods

### 2.1. Study Design and Population

This study is a single-center, retrospective observational analysis conducted at the Breast Surgery Unit of San Martino Hospital in Genoa, Italy. It focused on women aged 80 years and older who were diagnosed with either HER2+ or TNBC and treated surgically between March 2016 and October 2024 (*n* = 39). The study aimed to evaluate the outcomes of omitting SLNB in this elderly population, a group often underrepresented in clinical trials.

Eligible patients included those with a histologically confirmed diagnosis of HER2+ or TNBC who were clinically node-negative (cN0) based on axillary ultrasound assessment. Preoperative axillary ultrasound was performed in all patients using high-frequency linear transducers (≥10 MHz) in the supine position with the ipsilateral arm abducted, and radiologists assessed nodal morphology (cortical thickness, hilum preservation, shape, size). Any suspicious findings were documented, and if indicated, additional targeted imaging or biopsy was performed. All participating radiologists adhered to the same standardized criteria to minimize interobserver variability.

All patients underwent definitive surgical management, which included either breast-conserving surgery (quadrantectomy) or mastectomy, without any form of axillary surgery. To the purpose of outcome evaluation, only patients with at least one year of follow-up data were included in the survival analyses.

Exclusion criteria were defined to maintain a homogeneous study population and to reduce potential confounding factors. Specifically, we excluded patients younger than 80 years, those with luminal breast cancer subtypes, patients with clinically positive axillary lymph nodes, individuals who underwent axillary surgical procedures, and those with incomplete tumor staging or missing follow-up data. These criteria were applied to focus the study on a well-characterized elderly cohort in which the potential benefits and risks of omitting axillary surgery could be meaningfully assessed.

All eligible and consenting patients were included. Patients who met diagnostic inclusion criteria but elected to undergo SLNB were excluded, as were patients requiring SLNB or ALND based on diagnostic findings or those with a prior history of these procedures.

Patients were enrolled consecutively as part of routine clinical care, without randomization or experimental intervention. Informed consent for the use of medical data for research purposes was obtained during preoperative evaluations. Throughout the study, patient privacy and confidentiality were rigorously maintained, with all data anonymized and managed in compliance with General Data Protection Regulation (GDPR) guidelines.

### 2.2. Data Collection

Comprehensive data were retrospectively collected from the hospital’s electronic medical records and cross-verified by two independent reviewers to ensure accuracy. Collected variables included patient demographics (age at diagnosis), tumor characteristics such as size, histological grade, receptor status, proliferation index (Ki-67), and overall tumor biology (HER2+ or TNBC). Treatment-related information included details of the surgical procedure (quadrantectomy or mastectomy) and any perioperative or postoperative complications, including wound infections, seroma, hematoma, lymphedema, or functional limitations affecting arm mobility.

Oncological outcomes recorded included locoregional recurrence, distant metastasis, overall survival (OS), and disease-free survival (DFS). Follow-up information was obtained from routine outpatient visits and imaging studies.

### 2.3. Definitions

Overall survival (OS) was defined as the interval between the date of surgery and the date of death from any cause. Patients still alive at the data cut-off (7 April 2025) were censored at that date.

Disease-free survival (DFS) was defined as the time from surgery to the first documented breast cancer recurrence (local, regional, or distant) or death from any cause. Patients without an event were censored at the end of follow-up (7 April 2025).

Tumor subgroups were defined according to standard pathological and biomarker criteria. Tumor size categories were defined as ≤pT1c versus ≥pT2, and proliferation index was categorized as low (Ki-67 ≤ 25%) versus high (Ki-67 > 25%) for stratified analyses.

### 2.4. Statistical Analysis

Survival analyses were conducted using Kaplan–Meier methods to estimate OS and DFS, with survival curves compared using the log-rank test. Hazard ratios (HRs) and 95% confidence intervals (CIs) were calculated using Cox proportional hazards regression models to evaluate the association of clinical and pathological factors with survival outcomes.

Subgroup analyses were performed to investigate potential differences based on tumor biomarker subtype (HER2+ vs. TNBC), tumor size (≤pT1c vs. ≥pT2), and Ki-67 proliferation index (≤25% vs. >25%). All statistical tests were two-sided, and a *p*-value < 0.05 was considered statistically significant. Statistical analyses were conducted using R software (version 4.2.2; R Foundation for Statistical Computing, Vienna, Austria). Survival analyses, including Kaplan–Meier estimates and Cox proportional hazards models, were performed using the survival package (version 3.8-3) [12]. Survival curves were visualized using the survminer package (version 0.5.1) [13] and ggplot2 (version 4.0.0) [14]. All analyses were performed according to standard statistical practices and guidelines.

## 3. Results

### 3.1. Patient Characteristics

A total of 39 women aged ≥80 years (mean age at surgery: 85.8 ± 4.0 years; range: 80.3–97.4) were included. Among them, 28 (71.8%) had HER2+ tumors and 11 (28.2%) TNBC. All were clinically node-negative and underwent surgery without SLNB or ALND. About 47.1% had a prior history of breast cancer (ipsilateral or contralateral).

The median tumor size was 17 mm (IQR: 12–24; mean: 20.7 mm, SD: 18.4). Most were high grade (59% G3), and the majority were classified as pT1c (48.7%) or pT2 (28.2%). The median Ki-67 index was 27.0% (IQR: 21.9–58.1), with 47.2% ≤25%.

The surgical approach was mostly conservative, with quadrantectomy performed in 27 cases (69.2%).

During follow-up, five recurrences occurred (12.8%): three local (managed with mastectomy) and two axillary (managed with ALND). The median follow-up was 36.5 months (IQR: 21–49), based on all 39 patients.

Only two complications arose (5.1%): a wound dehiscence (which required another surgery) and a wound infection (which was successfully managed with antibiotics and outpatient care).

The characteristics of the cohort are summarized in Table 1.

### 3.2. Survival Outcomes

**Figure 1:**
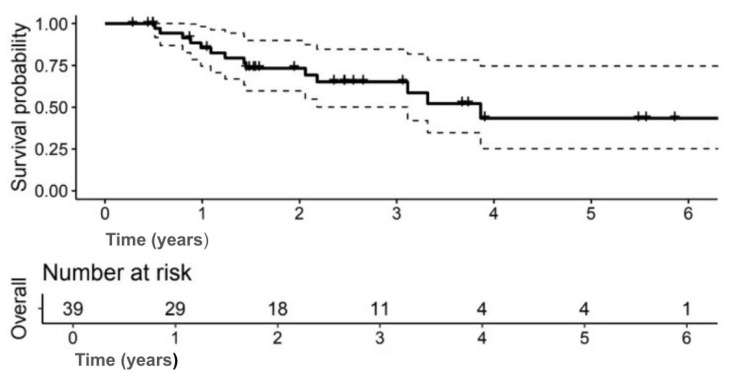
Kaplan–Meier curve showing overall survival (OS) for the study cohort. (Solid lines indicate survival probability, and dashed lines indicate 95% confidence intervals (CI). Tick marks represent censored events. Numbers at risk are shown below the *x*-axis).

**Figure 2:**
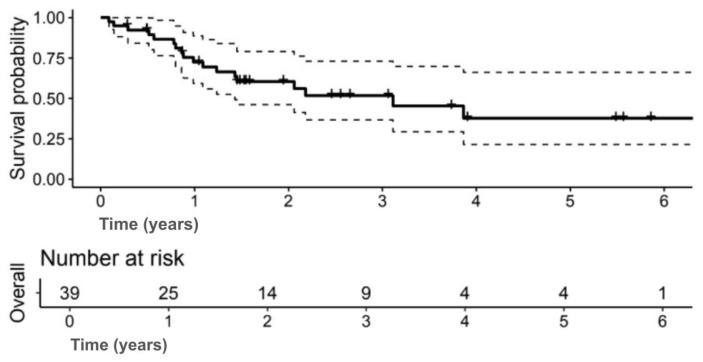
Kaplan–Meier curve showing disease-free survival (DFS) for the study cohort. (Solid lines indicate survival probability, and dashed lines indicate 95% CI. Tick marks represent censored events. Numbers at risk are shown below the *x*-axis).

The median OS was 3.9 years (95% CI: 3.1–NA); 5-year OS: 43.4% (95% CI: 25.3–74.6) (Figure 1). The 5-year DFS was 37.7% (95% CI: 21.5–66.2) (Figure 2).

### 3.3. Subgroup Analyses

**Figure 3:**
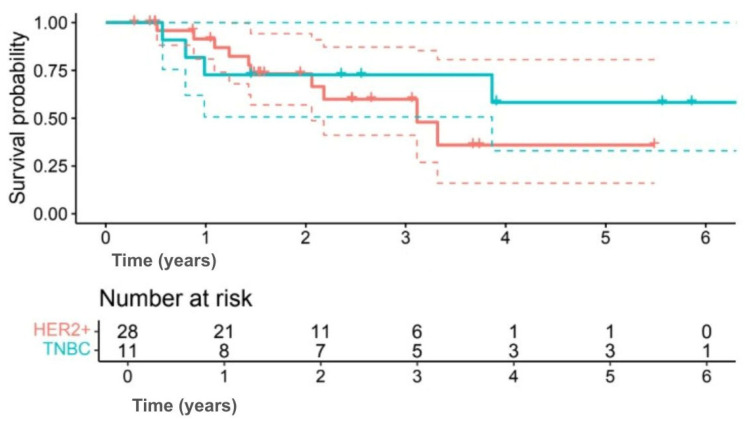
Kaplan–Meier OS curves comparing triple-negative breast cancer (TNBC; green line) and HER2-positive (HER2+; red line) patients. (Solid lines indicate survival probability, and dashed lines indicate 95% CI. Tick marks represent censored events. Numbers at risk are shown below the *x*-axis. Survival differences were evaluated using the log-rank test (*p* = 0.414)).

**Figure 4:**
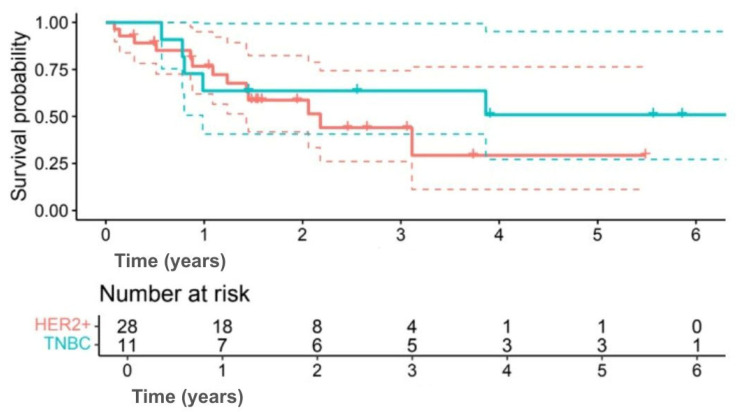
Kaplan–Meier DFS curves comparing TNBC (green line) and HER2+ (red line) patients. (Solid lines indicate survival probability, and dashed lines indicate 95% CI. Tick marks represent censored events. Numbers at risk are shown below the *x*-axis. Survival differences were evaluated using the log-rank test (*p* = 0.402)).

Exploratory subgroup analyses suggested differences in survival outcomes, although none were statistically significant. Among subtypes, 5-year OS was 58.2% for TNBC compared with 35.9% for HER2+ (HR 0.60, 95% CI 0.17–2.09; *p* = 0.414) (Figure 3), and 5-year DFS was 50.9% versus 29.4%, respectively (HR 0.63, 95% CI 0.19–2.12; *p* = 0.402) (Figure 4). Interpretation was limited by small sample numbers.

**Figure 5:**
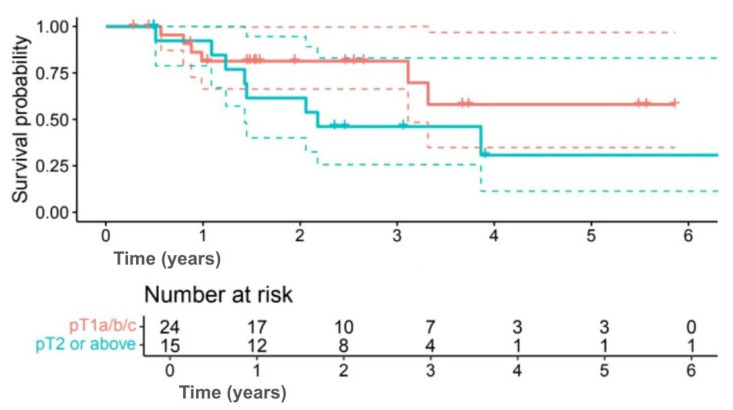
Kaplan–Meier OS curves comparing tumor sizes ≤pT1c (red line) and ≥pT2 (green line). (Solid lines indicate survival probability, and dashed lines indicate 95% CI. Tick marks represent censored events. Numbers at risk are shown below the *x*-axis. Survival differences were evaluated using the log-rank test (*p* = 0.118)).

**Figure 6:**
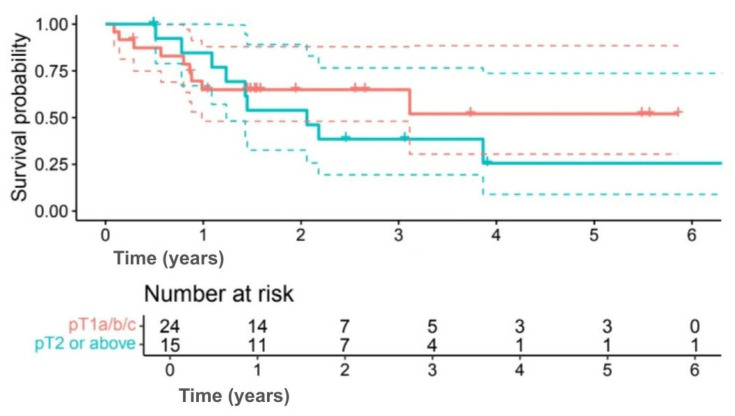
Kaplan–Meier DFS curves comparing tumor sizes ≤pT1c (red line) and ≥pT2 (green line). (Solid lines indicate survival probability, and dashed lines indicate 95% CI. Tick marks represent censored events. Numbers at risk are shown below the *x*-axis. Survival differences were evaluated using the log-rank test (*p* = 0.133)).

Tumor size showed similar results, with 5-year OS of 58.1% for tumors ≤ pT1c compared with 30.8% for tumors ≥ pT2 (*p* = 0.118) (Figure 5), and DFS of 51.9% versus 25.6% (*p* = 0.133) (Figure 6).

**Figure 7:**
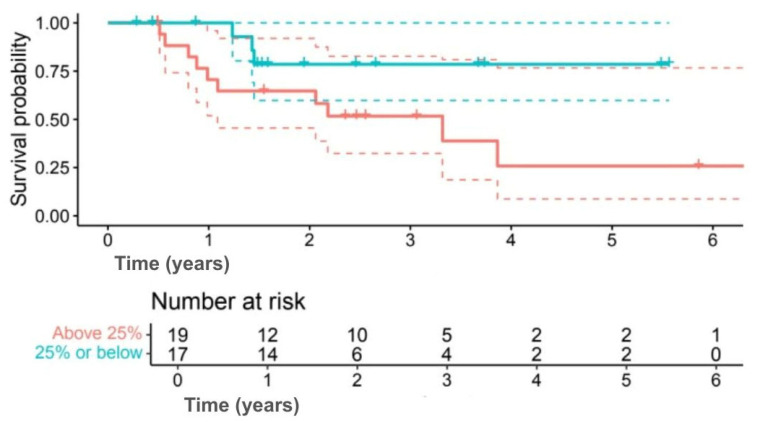
Kaplan–Meier OS curves comparing Ki-67 ≤25% (green line) and >25% (red line). (Solid lines indicate survival probability, and dashed lines indicate 95% CI. Tick marks represent censored events. Numbers at risk are shown below the *x*-axis. Survival differences were evaluated using the log-rank test (*p* = 0.080)).

**Figure 8:**
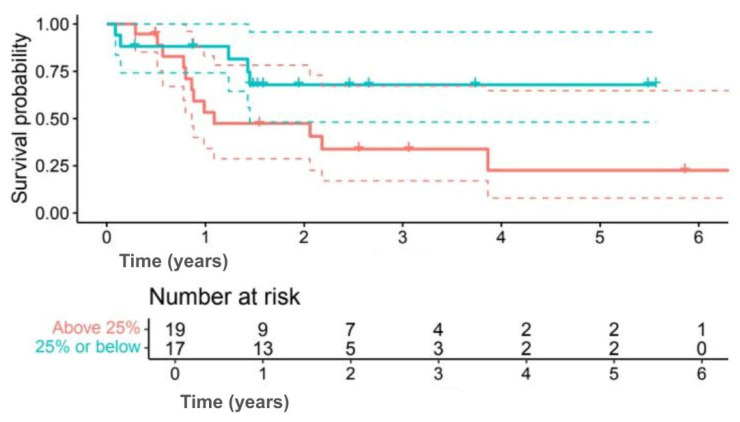
Kaplan–Meier DFS curves comparing Ki-67 ≤25% (green line) and >25% (red line). (Solid lines indicate survival probability, and dashed lines indicate 95% CI. Tick marks represent censored events. Numbers at risk are shown below the *x*-axis. Survival differences were evaluated using the log-rank test (*p* = 0.092)).

Analysis by Ki-67 index indicated numerically better outcomes for lower proliferation. Five-year OS was 78.6% for Ki-67 ≤ 25% compared with 25.9% for Ki-67 > 25% (*p* = 0.080) (Figure 7), while 5-year DFS was 67.9% versus 22.6% (*p* = 0.092) (Figure 8). Although outcomes consistently favoured the low Ki-67 group, differences were not statistically significant.

## 4. Discussion

Our results align with those of previous studies and give no indication that SLNB omission negatively affects OS or DFS. To contextualize these findings, we reviewed relevant literature examining older breast cancer patients (≥70–75 years) who underwent SLNB. Gu et al. (2022) showed no significant DFS difference in patients >70 years based on SLNB status [2]. Chung et al. demonstrated that SLNB in women ≥65 years often escalates treatment without survival benefit [15]. Reimer et al., analyzing over 5000 women in the INSEMA trial confirmed no OS disadvantage when SLNB was omitted in low-risk older patients [16]. Collectively, these data emphasize that tumor biology and comorbidities may outweigh nodal status in determining prognosis [17]. In this context, our results suggest that omission of SLNB may be safe even in higher-risk subtypes.

Notably, TNBC patients in our series did not show worse outcomes than HER2+ cases, though this must be interpreted with caution given the very small numbers and curve crossing in survival analyses.

Emerging literature also indicates that systemic therapy plays a dominant role in outcomes for these subtypes [18]. For HER2+ disease, large databases show nodal positivity is exceedingly rare among those achieving pathological complete response after neoadjuvant HER2-targeted therapy, limiting the incremental value of SLNB [19]. For TNBC, studies in older patients highlight that undertreatment (especially chemotherapy omission) is associated with worse outcomes, while aggressive local therapy confers limited benefit in very elderly patients [11,20]. Together, this supports the notion that when systemic therapy is already indicated, the additional prognostic contribution of SLNB may be limited in this age group.

It is also important to consider the natural life expectancy of women in this age group. For example, in many high-income countries, women aged 85–90 years have a median life expectancy of approximately 5–7 years [21]. This reality underscores the necessity of balancing the potential benefits of invasive staging procedures against the risks of complications, recovery time, and impact on overall well-being, particularly when the likelihood of breast cancer–related mortality may be lower than competing risks.

We acknowledge several limitations in our study, including its retrospective design, single-center nature, and relatively small sample size (reflecting strict age-based eligibility). Importantly, the absence of an internal control group of elderly patients who underwent SLNB precludes causal inference; comparisons were drawn from existing literature. Furthermore, cause-of-death data were unavailable, preventing analysis of cancer-specific survival. As for the results, we recognize that subgroup analyses were underpowered; they are presented as exploratory data. Hazard ratios are reported descriptively but should not be overinterpreted.

Nevertheless, we believe that these findings provide support for personalized, biomarker-driven surgical strategies in elderly breast cancer patients, highlighting the potential to tailor axillary management to individual risk profiles and optimize quality of life without compromising oncological outcomes.

## 5. Conclusions

Omitting SLNB in very elderly patients (≥80 years) with HER2+ or TNBC and no clinical nodal involvement may represent a reasonable option in carefully selected cases. This strategy may be particularly appropriate for patients with smaller tumors and low Ki-67 proliferation indices, where the risk of axillary metastasis is relatively low. By reducing the extent of surgical intervention, clinicians can ethically minimize the risk of procedure-related complications, including lymphedema, wound infections, and impaired arm function, while still maintaining effective oncological outcomes for this vulnerable patient population.

Importantly, the decision to omit SLNB should be made following a comprehensive assessment by a multidisciplinary team, accounting for tumor biology, patient comorbidities, and overall functional status. Such individualized planning ensures that the benefits of de-escalated surgery are balanced against potential oncological risks.

Our results should be interpreted as preliminary rather than definitive. Nonetheless, they highlight the potential role of individualized surgical de-escalation strategies in elderly patients.

To further validate these observations and support evidence-based clinical practice, larger multicenter studies are needed. These studies should aim to confirm the safety and long-term outcomes of omitting SLNB in elderly patients with high-risk breast cancer subtypes, ultimately contributing to the development of more personalized and patient-centered approaches to breast cancer management in older populations.

## Figures and Tables

**Table 1:** Table summarizing cohort characteristics (n = 39).

Characteristic	N (%) or Median [Range]
Age at surgery (years)	Median 86.3 [80.0–97.4]
Sex	Female: 39 (100%)
Tumor biological subtype	
Triple-negative	11 (28.2%)
HER2+	28 (71.8%)
Tumor histological grade	
G1	1 (2.6%)
G2	17 (43.6%)
G3	21 (53.8%)
Tumor staging	
≤pT1c	25 (64.1%)
≥pT2	14 (35.9%)
Proliferation index (Ki-67)	
≤25%	17 (43.6%)
>25%	22 (56.4%)
Surgery	
Conservative	27 (69.2%)
Mastectomy	12 (30.8%)
Recurrences	5 (12.8%)
Complications	2 (5.1%)

## Data Availability

Data supporting reported results are available in institutional database.
